# Designing a respectful maternity care guideline: a multiphase study

**DOI:** 10.1186/s12978-022-01389-8

**Published:** 2022-03-28

**Authors:** Khadije Hajizadeh, Maryam Vaezi, Shahla Meedya, Sakineh Mohammad Alizadeh Charandabi, Mojgan Mirghafourvand

**Affiliations:** 1 Midwifery Department, Tabriz University of Medical Sciences, Tabriz, Iran; 2 Alzahra Teaching Hospital, Tabriz University of Medical Sciences, Tabriz, Iran; 3 South Asia Infant Feeding Research Network (SAIFRN), School of Nursing, Faculty of Science, Medicine and Health, University of Wollongong, Wollongong, Australia; 4 Department of Midwifery, Faculty of Nursing and Midwifery, Tabriz University of Medical Sciences, Tabriz, Iran; 5 Social Determinants of Health Research Center, Tabriz University of Medical Sciences, Tabriz, Iran

**Keywords:** Respectful maternity care, Guideline, Delphi technique, Iran

## Abstract

**Background:**

There is no comprehensive guideline for respectful maternity care (RMC) promotion in Iran. This study aimed to design a RMC guideline based on a multiphase study.

**Methods:**

In this multiphase mixed-methods study, recommendations were made for RMC promotion through the data obtained from Phase I (i.e., the quantitative section with a cross-sectional design), Phase II (i.e., the qualitative section with a content analysis method), and Phase III (i.e., focus group discussions with birth attendants as well as opinions of the specialized panel through the Delphi technique). The composed recommendations were then analyzed and finalized by relevant specialists in terms of execution capacity, approvability, and cost-effectiveness within the current context of Iran. Eventually, the resultant guideline were evaluated and approved by two members of the research team specializing in the research area in accordance with the Appraisal of Guideline for Research and Evaluation (AGREE).

**Results:**

The results of this multiphase study led to 80 recommendations for RMC promotion. The recommendations were classified as eight areas called recommendations for the pregnancy period, recommendations for the labor period and delivery, recommendations for the neonatal period, occupational recommendations, supervision recommendations, national policy recommendations, recommendations for training students and staff, and general public recommendations.

**Discussion:**

Based on the outcomes of disrespect and abuse, it is recommended to provide comprehensive guideline for policymakers and planners to formulate plans through the RMC promotion approach. Healthcare service policymakers can use this guideline to design some interventions to meet women’s financial, psychological, and legal needs.

## Plain language summary

Disrespect and abuse in the delivery room is associated with negative experience of delivery and poor maternal care quality index. Also, disrespect and abuse is the main barrier to achieving maternal health outcomes. In spite of the considerable achievements in maternal and child health, there is still a large number of maternal and neonatal mortality worldwide. It seems that disrespect and abuse is a key potential obstacle hindering access to delivery facilities and skilled care providers. To achieve the sustainable development 2030 goals developed by the World Health Organization (WHO) (Goal 3.1: Ensure health lives and promote well-being for all at all ages: reduce the global maternal mortality ratio to less than 70 per 100,1000 live birth), stakeholders and relevant institutions should consider respectful pregnancy and delivery care services as a key solution to reducing maternal mortality. Nevertheless, there is no comprehensive guideline with regards to respectful maternity care in the WHO or provided by other countries. In this multiphase mixed-methods study, recommendations were made for respectful maternity care promotion through the data obtained from Phase I (i.e*.*, the quantitative section with a cross-sectional design), Phase II (i.e., the qualitative section with a content analysis method), and Phase III (i.e., focus group discussions with birth attendants as well as opinions of the specialized panel through the Delphi technique). The composed recommendations were then analyzed and finalized by relevant specialists in terms of execution capacity, approvability, and cost-effectiveness within the current context of Iran. The results of this multiphase study led to 80 recommendations for RMC promotion. The recommendations were classified as eight areas called recommendations for the pregnancy period, recommendations for the labor period and delivery, recommendations for the neonatal period, occupational recommendations, supervision recommendations, national policy recommendations, recommendations for training students and staff, and general public recommendations.

## Background

Labor and especially delivery are sensitive and vulnerable periods. Pain, insecurity, threat and exposure to situation are the cause of vulnerability of women in labour and delivery [1, 2]. Typically, in delivery the concept of “safe motherhood” is limited to physical safety, while delivery has also deep cultural and personal significance for both the women and their family. Thus, the concept of safe motherhood should transcend beyond issues such as prevention from disease or mortality, and other concepts including respecting human rights such as respecting independence, esteem and respect, choices and preferences, as well as solidarity with women should also be considered [3].

Lack of privacy, which includes disregard for visual privacy, lack of confidentiality, insufficient attention to cultural and religious issues in terms of gender differences between parturient women and medical staff, upset about the presence of additional medical staff in the woman's bedside and multiple examinations should be limited. World Health Organization (WHO) has emphasized limitation of vaginal examinations to the degree of necessity or every four hours to diagnose long labour [4].

Every woman is entitled to receiving quality healthcare with characteristics including respect, free of violence and discrimination, with sufficient health information in pregnancy, labour and delivery [5]. Health equity is one of the components of respectful maternal care in which pregnant women should not be discriminated against by race, or cultural or social differences [3, 6]. Inequities are created when barriers prevent individuals and communities from accessing these conditions and reaching their full potential. Health disparities are one way we can measure our progress toward achieving health equity [7].The disrespect and abuse (D&A) outcomes are known as public health concerns worldwide [5, 8].

D&A in the delivery room is associated with negative experience of delivery [9] and poor maternal care quality index [10]. Also, D&A is the main barrier to achieving maternal health outcomes [10]. In spite of the considerable achievements in maternal and child health, there is still a large number of maternal and neonatal mortality worldwide. It seems that D&A is a key potential obstacle hindering access to delivery facilities and skilled care providers [11]. To achieve the sustainable development 2030 goals developed by united nations (Goal 3.1: Ensure health lives and promote well-being for all at all ages: reduce the global maternal mortality ratio to less than 70 per 100,1000 live birth) [12], stakeholders and relevant institutions should consider respectful pregnancy and delivery care services as a key solution to reducing maternal mortality. Nevertheless, there is no comprehensive guideline with regards to respectful maternity care (RMC).

In a study by Down et al. [13] to explore the efficacy of respectful maternity care policies for women utilizing delivery services, the evidence with moderate reliability indicated that the interventions (such as training change of attitudes and values, launching quality improvement teams, mentorship program, holding educational workshops in the community, mediation and conflict resolution, and consultation with those suffering from abuse and disrespect, training communication skills, establishing sympathy, support during the delivery, holding educational sessions during pregnancy, and familiarizing women with their rights) could enhance positive maternal experiences. Furthermore, respect-associated policies would enhance quality care as well [13].

A guideline is a written plan which specifies the methods and standards that should be followed in examination or provision of care for a special condition [14]. In this paper, determination of RMC status during delivery in the quantitative phase, and interpretation of the perception of women who have given birth about the determining factors and aspects of RMC in the qualitative phase, and combination of the results of the quantitative and qualitative phases in the combination stage could be useful in providing a guideline for promoting RMC in maternity wards. Implementation of this guideline would eventually contribute to enhancing the quality of care and reducing D&A in delivery. Since there is no comprehensive guideline in the World Health Organization (WHO) or provided by other countries for RMC, this study is the first of its type designed to develop RMC guideline in Iran based on a multiphase study.

## Methods

### Study design

This study is a mixed-methods study with sequential explanatory approach that was carried out from May 2019 to March 2020 in Tabriz, Iran. Since there is no comprehensive guideline in Iran for RMC, this study is the first of its type designed to develop RMC guideline in Iranian women based on a multiphase study. This study was performed after approval of the proposal and receiving ethics code from the ethics committee of Tabriz University of medical sciences (IR.TBZMED.REC.1398.202).

The protocol of this study has previously been published [15]. In this study, three aims were considered, which were: 1- To determine the status of D&A and RMC; 2- To explore the Iranian mother's perspectives about determinants of disrespect and abuse during childbirth and 3- To provide the guideline for promoting RMC.

In this multiphase mixed-methods study, recommendations were made for RMC promotion through the data obtained from Phase I (i.e., the quantitative section with a cross-sectional design), Phase II (i.e., the qualitative section with a content analysis method), and Phase III (i.e., a focus group discussion with birth attendants as well as opinions of the specialized panel through the Delphi technique).

### First phase

The first phase was a cross-sectional study performed to determine the status of RMC as well as the predictive factors of D&A. Sampling was conducted in the postpartum ward of public (Alzahra, 29 Bahman and Taleghani) and private (Behbood, Nor-e-Nejat, and Shahriyar) hospitals in Tabriz, Iran. In this phase, 334 postpartum women were selected in proportion to the number of births in these hospitals 3 months prior to the study.

In this phase, personal and social demographic characteristics, RMC and D&A questionnaires were used, which were completed 6–18 h post-delivery. Multivariate logistic regression test was employed to determine the D&A predictors [16].

### Second phase

The second phase was qualitative study based on content analysis with a conventional approach. The advantage of a conventional approach in qualitative content analysis is the ability to gather data directly from study participants without imposing predetermined categories and previous theoretical assumptions. In this method, knowledge generated from content analysis is based on the unique views of the participants and is rooted in text data [17].

Based on the results of the quantitative phase with regards to the total mean score of RMC among women who had given birth, those who had acquired 10% of the lower and upper limit of the total RMC score (the extreme cases) were considered as the participants of qualitative study. In this phase, interviews were done with 12 mothers with a wide range of ages, levels of education, and social classes. The interviews began with general questions such as “How was the day that you gave birth to your child?” “What memories do you have from that day?” “In your opinion, what are the factors could cause D&A during childbirth?” “In your opinion, how can D&A affect your experience?”.

Semi-structured and in-depths interviews were conducted by the KH (first author) in comfortable and natural place (mother’s home or health centre). The interviewer took notes in addition to the audio recording by an mp4 device during the interview. The sentences and phrases (meaning units) were coded. The similar codes were merged and those that indicated related concepts were categorised together and the subcategories were formed. Next, the subcategories were compared and the similar ones were inductively grouped in one category. Interviews continued until data saturation was reached. The duration of interviews varied from 30 to 90 min. In-depths interviews at different times and places and maximum diversity of participants were used to validate the findings [18].

### Third phase

The results obtained from the quantitative and qualitative stages were merged with categories of focus group discussion with birth attendants in the guideline, and prepared as recommendations. Next, Delphi technique was developed with participation of a specialized panel for assessing the guideline. Specifically, in the final stage, a set of recommendations was localized according to the conditions of Iran (in terms of executability, approvability, and cost-effectiveness) according to the relevant specialists. The stages of this phase were performed as follows:

### Focus group discussion

A group session of 12 attendants and delivery care providers was formed from two educational hospitals of Alzahra and Telaghani in Tabriz city in the conference room of Alzahra medical-educational center during 90 min in March 2020. The attendants and delivery care providers who had a willing to describe their perception about determinants of D&A and RMC during childbirth were selected for Focus group discussion.

A member of the team leads the group and asks probing questions and guides participants. Interview guide was developed by literate review and the results of qualitative and quantitative phases. The discussion began by a general RMC question, and then the participants were asked to express their opinion about the factors affecting RMC and D&A. Also during the discussion, exploratory questions were posed by the leader of the team so that the subject would be discussed more in detail. Alternatively, the participants were asked to mention an example or case so that their idea would be understood more clearly. Concurrent with expression of opinions, the discussed points were recorded and transcribed.

### Delphi technique

This technique was performed in the following five stages [19]:

- *Stage I* The selection, reason, and rationale of study were explained to the specialist panel for participation in the activities. Selection of the specialists was purposeful, whereby the individuals who had experience of providing clinical services to women and were scholars of the issues related to respectful maternity care were chosen. The specialties of this panel included midwifery (4 experts), gynecology (3 experts), and reproductive health (5 experts) were selected from faculty members of different cities across Iran (Tabriz, Mashhad, Ahwaz, Ardabil and Hamadan). The specialties of this panel were selected from scientific areas.
- *Stage II* Each specialist was asked to express their opinion about the predetermined issues or problems. At this stage, three open questions were posed whereby the experts were requested to present written responses about those questions. The questions were as follows:
  - What factors can promote RMC?
  - What factors can be effective in facilitation of D&A?
  - What factors can inhibit D&A?
- *Stage III* The responses of stage II were collected. Duplicate responses were eliminated and some responses with similar concepts were merged. The expert responses and the results of qualitative plus quantitative phases along with the items of focus group discussion with birth attendants were prepared as statements to be used in the next round.
- *Stage IV* At this stage, the expert members were requested to express their agreement or disagreement about each statement regarding its impact on respectful maternity care as well as executability and cost-effectiveness. The responses had been graded based on a Likert scale ranging from absolutely agree (score 4) to absolutely disagree (score 0), and non-executable.
- *Stage V* The statements for which the experts had not reached consensus, were presented again for the next stage. The consensus was considered agreement of 70% and above.

The prepared guideline was assessed and confirmed using Appraisal of Guideline Research and Evaluation (AGREE) standard instrument by two members of the research team with specialty in the subject area. In fact, AGREE is a standard instrument for criticizing clinical practice guideline. It deals with assessing both the quality of the way the issues are presented and the quality of some aspects of recommendations. Indeed, it investigates the possibility of success of the guideline in achieving the desired behavioral outcomes. AGREE checklist has six dimensions exploring the guideline in terms of goals, involving the policymakers, literature review, clarity and transparency of the guideline, its executability, and conflicts of interest [20].

## Results

### First phase: quantitative phase

A total of 334 women were included in this phase of study. Approximately half of them (161; 48.5%) aged between 26 and 35 years. The majority (319; 95.5%) were stay-at-home mothers, with moderate economic status (256; 76.6%). Less than half of the participants had high school education (146; 43.7%), and 42.2% were first-time mothers.

In this phase of the mixed-method study, the prevalence of D&A in Iran was reported 75.7%, with the major type of D&A being related to not allowing selection of the position (44.3%) and immobility in labor (42.5%). Further, the results of this phase indicated that delivery during the night (aOR 3.07; 95% CI 1.61 to 5.88) increased the likehood of D&A. However, delivery by a private physician or midwife (aOR 0.05; 95% CI 0.02 to 0.12), as well as presence of the spouse in waiting room (aOR 0.32; 95% CI 0.11 to 0.88) can reduce D&A in the delivery room. The mean (standard deviation) of the total RMC score was 63.42 (19.28) from the obtainable score range of 0 to 100 [16].

### Second phase: qualitative phase

A total of 12 women were included in this phase of study. Two-thirds of participants (8; 66.6%) aged between 26 and 35 years and more than half of them were housewives (7; 58.3%) and have moderate economic status (7; 58.3%). Less than half of them had high school education (5; 41.6%).

After analyzing the transcripts of interviews, 38 subscales and 11 main categorized were found including:

1. Physical abuse was made of two sub-categories of physical harassment and use of force.
  *They even wanted to beat me. They pounded my legs to open them for examination.” (Participant 4)*
2. Psychological violence was made up of four sub-categories: verbal abuse, mocking the parturient women, insulting the parturient women, and threatening and intimidating the parturient women.
  *“They commanded me angrily ‘open your legs!.” (Participant 8)*
3. Discrimination between women including sub-categories of discrimination based on personal-social characteristics, and discrimination based on having relations at the hospital.
  *“The midwife insult rural woman with bad words.” (Participant 8)*
4. Violating the privacy was made up of four sub-categories: violation of personal space and visual privacy, non-confidentiality, insufficient attention to cultural and sharia principles in the gender difference between the parturient woman and the caregivers, presence of excessive number of caregivers, and excessive examination.
  *“The doctors repeatedly came and examined me and I was very uncomfortable.” (Participant 11)*
5. Unfulfilled needs was made of four sub-categories of negligence of providing comforting services and environment, negligence of the nutritional needs, limitation of the presence of husband or attendant family member, and negligence of the parturient woman’s request for analgesics.
  *“I was very thirsty after my childbirth. I didn’t have dinner and I was hungry too.” (Participant 9)*
6. Lack of participation in decision-making including sub-categories of limitation of movement and change of position, and non-participation in the decisions related with the women.
  *“They didn’t let me move. From midnight to 9 A.M. a woman feels a lot of pressure on her back at childbirth.” (Participant 1)*
7. Neglecting the pregnant women was made up of three sub-categories including insufficient and improper provision of information and instruction related to the women’s needs, delay in care, and lack of correct follow-up.
  *“Midwife did not even give a yes or no. I wish she said ‘yes’ even with a bad tone.” (Participant 12)*
8. No sympathy was made of three sub-categories of negligence of women’s emotional needs, lack of understanding, and negative mood.
  *“The doctor constantly said, ‘natural childbirth may not be possible for you, it’ll be a C-section then.” (Participant 3)*
9. Deficit of human and nonhuman resources was made up of five sub-categories: shortage of equipment and amenities, shortage of caregiving personnel, personnel work overload, unpleasant condition and improper services of the ward, and insufficient physical space.
  *"The sheets would get dirty and the assistant nurses didn’t regularly change the sheets. The bathroom was also dirty.” (Participant 6)*
10. Unpleasant psychological atmosphere of the center was made up of two sub-categories including anxiety-inducing environment, and fear-inducing environment.
  *“When I went to the hospital they blamed me for Enoxaparin injection and said, ‘You’re going to bleed a lot.” (Participant 9)*
11. Facilitators of disrespect and abuse consisted of five sub-categories including unskilled students and caregivers, personnel fatigue and drowsiness, lack of personnel qualifications, personnel psychological problems, and disorderliness.
  *“At midnight, midwives were very abusive. I think that must’ve been because of fatigue and drowsiness.” (Participant 10)*

### Findings of the specialized panel

The characteristics of the expert members are presented in Table 1. In the first round, the expert opinions were collected with regard to the aspects and factors of D&A or RMC.

**Table 1:** Characteristics of experts in the Delphi technique (n = 12)

Participant	Sex	Degree (s)	Academic degree	Years of clinical experience	Area of specialty
1	Female	MD, Fellowship	Assistant professor	7	Obstetrics, MFM^a^
2	Female	MD, Fellowship	Associate professor	29.5	Obstetrics, MFM
3	Female	MD, Fellowship	Assistant professor	15	Obstetrics, MFM
4	Female	MSc	Instructor	26	Midwifery
5	Female	MSc	Assistant professor	30	Midwifery
6	Female	MSc	Instructor	27	Nursing of maternal and child health
7	Female	MSc, PhD	Associate professor	26	Health reproductive
8	Female	MSc, PhD	Assistant professor	23	Health reproductive
9	Female	MSc, PhD	Assistant professor	5	Health reproductive
10	Female	MSc, PhD	Instructor	21	Health reproductive
11	Female	MSc, PhD	Instructor	23	Health reproductive
12	Female	MSc, PhD	Associate professor	26	Health reproductive

^a^Maternal Fetal Medicine

After coding and classifying the opinions of all experts (n = 12), 198 recommendations were obtained. After merging and removing duplicate items with the same concepts, 42 recommendations remained. Another 38 recommendations resulting from quantitative and qualitative results of the study plus focused group discussion with the birth attendants (n = 12) were also added to the 42 recommendations resulting from the expert opinion in the first round. Eventually 80 recommendations were prepared as statement for the second round. Consensus was achieved for all recommendations (Table 2).

**Table 2:** Final set of recommendations for improving of respectful maternity care in the Iran

Statement	Consensus type	Percentage of consensus	Responsible unit
Recommendations for pregnancy period			
1. Pregnant mothers should be trained in reasonable expectations	Completely agree/agree	88.33	Midwife, Physician
2. The patient charter of rights should be made available to pregnant women during visit and treatment stages	Completely agree/agree	75	Midwife, Physician
3. The pregnant women should participate in delivery preparation classes during her pregnancy and become familiar with her rights with regards to delivery	Completely agree/agree	100	Midwife, Physician
4. Before the delivery, some sessions should be held for pregnant mothers to establish trust with birth attendants, training maternal rights, and familiarization with different hospital ward	Completely agree/agree	91.66	Midwife, Physician
Recommendations for the labor, delivery and postpartum stages			
5. The number of birth attendants should be reasonable and standard (one-to-one system) in order to prevent the fatigue of healthcare teams and service providers, especially during night shifts	Completely agree/agree	91.66	Heads of the center
6. It is necessary to consider the privacy of parturient women and attention to cultural as well as religious issues regarding gender differences between the parturient women and healthcare teams at all hospitalization stages	Completely agree/agree	100	Midwife, Physician
7. Informed consent forms should be taken during examinations and interventions	Completely agree/agree	75	Midwife, Physician
8. Effective communications should be established between the midwife/physician and parturient women through simple methods in line with women’s cultural backgrounds	Completely agree/agree	91.66	Midwife, Physician
9. The hospital personnel and birth attendants should avoid reprehension, insult, threats, humiliation, mockery, and any improper or violent behavior	Completely agree/agree	100	Midwife, Physician and assistant nurse
10. The midwife/physician should have attention, sympathy, heart-warming, promising words (inducing positive spirit) and proper eye contact for the parturient women	Completely agree/agree	100	Midwife, Physician
11. The midwife/physician should introduce themselves to the parturient women and present their job descriptions	Completely agree/agree	91.66	Midwife, Physician and assistant nurse
12. All hospital employees should wear uniform costumes so that the parturient women will know their care providers	Completely agree/agree	100	Midwife, Physician and assistant nurse
13. The midwife/physician should respond to all patients’ questions attentively	Completely agree/agree	100	Midwife, Physician and assistant nurse
14. The midwife/physician/assistant should treat the parturient women and their companions (especially at initial admission) respectfully	Completely agree/agree	100	Midwife, Physician and assistant nurse
15. Upon the entrance of the parturient women, the system (a person with midwifery specialty) should be present to welcome and introduce the wards as well as hospital rules	Completely agree/agree	100	Midwife, Physician and assistant nurse
16. The parturient women’s necessary personal should be easily accessible to them	Completely agree/agree	91.66	Assistant nurse
17. The midwife/physician should inform the parturient women about their health status, that of the fetus, and that of their family (with no induction of apprehension)	Completely agree/agree	100	Midwife, Physician
18. The care provider should inform the parturient family about the maternal, fetal, and neonatal status	Completely agree/agree	91.66	Midwife, Physician
19. The hospital system should avoid providing improper, bad-colored, and old clothing for the parturient women	Completely agree/agree	91.66	Assistant nurse
20. It is necessary to provide the conditions for the presence of a personal midwife or physician alongside the parturient women	Completely agree/agree	75	Head of center
21. The necessary conditions should be provided for the presence of doula alongside the parturient women	Completely agree/agree	100	Head of center
22. The midwife/physician/assistant should avoid disputes, discussion, and whispering about the health status of women in labor (on mother’s bedside)	Completely agree/agree	100	Midwife, Physician and assistant nurse
23. Constant care should be provided for mothers (in the presence of accompanying physicians or midwives from early stages of pregnancy until delivery and postpartum periods)	Completely agree/agree	83.33	Head of center
24. Proper nutrition should be provided in labor as well as postpartum for the parturient women	Completely agree/agree	100	Assistant nurse
25. The physical atmosphere of the admission room, labor, and delivery should be suitable in terms of hygiene, light, temperature, and size	Completely agree/agree	100	Head of center
26. Proper well-fare equipment and facilities should be provided (e.g., suitable beds, hygienic products, and bath)	Completely agree/agree	100	Head of center
27. The atmosphere of the delivery center should be relaxing (through the use of vibrant colors, pleasant aroma, suitable decoration, and soothing music)	Completely agree/agree	91.66	Head of center
28. The emergency ward, labor room, delivery unit, and postpartum unit should be quiet	Completely agree/agree	83.33	Head of center
29. The parturient women should be involved in the decisions related to their care	Completely agree/agree	75	Midwife, Physician
30. During the labor and delivery, an informed and trained companion (e.g., a relative or a spouse) should be present alongside the parturient women, or they should have at least a short visit or contact with the parturient women	Completely agree/agree	100	Head of center
31. Discrimination between mothers should be avoided (experiencing differences in some personal and social characteristics or having an acquaintance in the hospital)	Completely agree/agree	91.66	Midwife, Physician and assistant nurse
32. Proper persistent and timely care should be provided	Completely agree/agree	100	Midwife, Physician and assistant nurse
33. Long stays at the hospital should be avoided before initiating the active phase of labor	Completely agree/agree	100	Midwife, Physician
34. The midwife/physician should explain the course of labor and different sections of delivery to the parturient woman (according to her understanding and language) and convince her completely	Completely agree/agree	91.66	Midwife, Physician
35. Frequent vaginal examinations should be avoided without acquiring permissions or proper justification of the parturient women	Completely agree/agree	100	Midwife, Physician
36. During the labor, the parturient women should be able to move freely or change positions	Completely agree/agree	91.66	Midwife, Physician
37. Pharmacological methods (e.g., venous or epidural opioids) or non-pharmacological methods (e.g., massaging, warm water shower and compress, breathing, and music) should be employed to alleviate pain during labor and delivery	Completely agree/agree	100	Midwife, Physician
38. The use of early oxytocin administration should be avoided	Completely agree/agree	100	Midwife, Physician
39. The inessential insertion of the bladder catheter should be avoided	Completely agree/agree	100	Midwife, Physician
40. The constant inessential cardiotocography for fetal health assessment should be avoided	Completely agree/agree	91.66	Midwife, Physician
41. The delivery status (e.g., sitting, upright, or lying positions) during the delivery (by providing prerequisites including trained attendants and proper environment) should be based on the women’s choice	Completely agree/agree	75	Midwife, Physician
42. Adequate anesthesia should be used during episiotomy and repair technique	Completely agree/agree	100	Midwife, Physician
43. Routine episiotomy should be avoided	Completely agree/agree	100	Midwife, Physician
44. The use of uterine fundal pressure to facilitate delivery in the second stage should be avoided	Completely agree/agree	91.66	Midwife, Physician
45. During the delivery, the parturient women should be encouraged and supported to exert the force of expulsion	Completely agree/agree	91.66	Midwife, Physician
Recommendation for care of the newborn			
46. The midwife/physician should inform the parturient women about the newborn’s status, the newborn’s gender, and the reason for delay in establishing skin-to-skin contact as well as breast-feeding postpartum	Completely agree/agree	100	Midwife, Physician
Occupational recommendations			
47. It is necessary to employ a healthcare provider team committed to ethical and professional principles and having both physical health and psychological health. The reason and significance of respectful maternity care should be explained at the onset of employment	Completely agree/agree	91.66	Head of center
48. Welfare facilities as well as financial, spiritual, psychological, legal needs plus occupational security of the midwife/physician should be fulfilled	Completely agree/agree	100	Head of center
49. It is essential to provide an incentive system which is a kind of serial and timely financial incentive plus promotion and motivation for individuals expressing respectful behavior	Completely agree/agree	100	Head of center
50. In case of repeating abuse and disrespect, financial punishment and documentation of misconduct should be in order	Completely agree/agree	83.33	Head of center
51. The system governing the hospital management should be based on dignity with an atmosphere filled with respect with no disputes with the low-rank staff	Completely agree/agree	100	Head of center
52. The working hours should be adjusted to the difficulty and occupational burnout of staff	Completely agree/agree	91.66	Head of center
53. In order to enhance the job satisfaction of midwives and gynecologists, discrimination should be avoided between the staff	Completely agree/agree	91.66	Head of center
54. Midwives and the heads of wards should provide services in different departments alternately in order to prevent depression or occupational burnout	Completely agree/agree	100	Head of center
Supervision recommendations			
55. The patient charter of rights should be publicized in posters so that it would be a constant reminder for studying and observing respectful maternity care	Completely agree/agree	100	Head of center
56. Pregnant mothers should be surveyed about the behavior and performance of the staff when they are leaving each ward. The results of such surveys should then be enforced	Completely agree/agree	91.66	Head of center
57. Induction of inhumane and unethical behavior of staff toward each other (e.g., exaggerated pride and sense of superiority over the patient) should be avoided	Completely agree/agree	75	Head of center
58. An employee with midwifery specialty should be present at different shifts of the delivery (especially during the night shift) frequently and unexpectedly. They should emphasize respect to the parturient women while understanding the midwife and parturient women’s conditions	Completely agree/agree	100	Head of center
59. There should be a system for accurately dealing with the complaints made by the patients and their companions	Completely agree/agree	91.66	Head of center
60. Improper and disrespectful behavior of the senior manager of the hospital should be modified and corrected so that it would not be induced to the low-rank staff	Completely agree/agree	91.66	Head of center
61. There should be regulatory and monitoring mechanisms for not only the physical performance of staff but also their types of behavior	Completely agree/agree	91.66	Head of center
62. The job descriptions of midwives should be corrected and clarified	Completely agree/agree	100	Head of center
63. A quality improvement team should be developed to assess and monitor disrespect and abuse in hospital	Completely agree/agree	100	Head of center
64. Consultation should be provided for mothers who have experienced abuse and disrespect	Completely agree/agree	91.66	Head of center
65. Executive laws should be formulated to observe respectful maternity care alongside some plans to monitor and review the procedure for enforcing the laws in hospitals constantly	Completely agree/agree	91.66	Head of center
National policy recommendations			
66. Provision of respectful services for mothers should be incorporated in the decisions made by policymakers as one of the main pillars of the healthcare system, which should also be further supervised with regard to its precise implementation	Completely agree/agree	91.66	Policy makers
67. There should be a national protocol of respectful maternity care with an emphasis on necessity, implementation, precise monitoring, frequent assessment, and removal of barriers to this protocol	Completely agree/agree	100	Policy makers
68. The grounds for implementing national protocols of respectful maternal care such as human and financial resources as well as hospitalization environment should be provided	Completely agree/agree	83.33	Policy makers
69. The rights of pregnant women and mothers should be taken into account by policymakers and community	Completely agree/agree	83.33	Policy makers
Recommendations for training students and staff			
70. Proper training in maternal rights, respectful maternity care, communication skills, and effective communication regulations should be incorporated in educational curricula at all medical and allied health fields	Completely agree/agree	91.66	Vice Chancellor For Education
71. For wrongdoers, frequent and compulsory training courses should be organized	Completely agree/agree	83.33	Vice Chancellor For Education
72. The behavioral challenges and interactions of gynecological specialists or residents with the midwife and vice versa should be corrected	Completely agree/agree	100	Vice Chancellor For Education
73. The educational approaches should be changed from merely care-based education with treatment approaches to respect-based care education	Completely agree/agree	100	Vice Chancellor For Education
74. Frequent educational courses should be held in order to promote communication and behavioral skills in clinical practice	Completely agree/agree	100	Vice Chancellor For Education
75. Classes in ethics should be held for the staff based on changes in attitudes and values	Completely agree/agree	100	Vice Chancellor For Education
76. Educational workshops should be provided by presenting posters and visual pamphlets in order to promote communication between the staff and mothers	Completely agree/agree	91.66	Vice Chancellor For Education
Recommendations related to the general public			
77. Respecting the other people’s rights in the society should be promoted widely so that it will become internalized as a cultural value over time	Completely agree/agree	100	Policy makers
78. The status of childbearing to maintain the family foundation should be internalized and honored in society	Completely agree/agree	91.66	Policy makers
79. The physical, psychological, mental, emotional, familial, and social advantages of pregnancy should be promoted publicly	Completely agree/agree	83.33	Policy makers
80. Educational workshops should be organized to familiarize mothers with their rights in society	Completely agree/agree	91.66	Policy makers

## Discussion

The results of this multiphase study led to presentation of 80 recommendations to promote RMC. The recommendations were categorized into eight areas including recommendations for pregnancy period, recommendations for the labor period and delivery, recommendations for the neonatal period, occupational recommendations, supervision recommendations, national policy recommendations, recommendations for training students and staff, as well as recommendations related to the general public.

The recommendations included providing the necessary conditions to familiarize women with different steges of delivery, birth attendants, and patient charter of rights. Other recommendations were introduced as receiving informed consent, instructing the hospital personnel and birth attendants to avoid reprehension, insult, threats, humiliation, mockery, improper treatment, and violent behavior. It was also recommended to provide attentive care and sympathy through heartwarming and promising words (infusing positive spirits) alongside eye contacts for parturient women with the appropriate proportion between the number of attendants for the provided care and others by promoting the staff skills in the delivery center, enhancing delivery centers in terms of a relaxing physical atmosphere, providing the necessary facilities and equipment, avoiding discrimination between the parturient women, keeping privacy through a curtain between women, providing the necessary conditions for phone contacts between women and their families, and using sufficient anesthesia during episiotomy and recovery.

Discrimination in the delivery room can be based on conditions such as age, economic, social status, literacy, ethnicity, urban or rural, or certain medical conditions. Discrimination against women can ultimately lead to abuse or insult to the mother. Studies on the relationship between abuse and demographic factors show that a number of factors such as adolescence age, low socioeconomic status, women with acquired immunodeficiency (HIV), were associated with higher D&A [21–23]. Changing discriminatory attitudes, beliefs and social norms and promoting the human rights of all women with respect for gender equality and non-violence are essential [24]. Culturally safe practice is predicated on relationships of respect and mutual trust. It is rights-based approach and supports principles of dignity, safety, autonomy, respect and empowerment [25].

Based on the findings in the present research, in the area of recommendations related to the pregnancy, through raising awareness and training pregnant women about the cares of pregnancy, labor, delivery, and postpartum, a positive impact can be created on RMC. Thus, use of the findings of this study, which have been presented for various stages and areas, can be employed to design training courses and to select proper educational methods in pregnancy classes. In this way, the parturient women is trained and made aware to participate in delivery preparation classes during their pregnancy and become familiar with their rights with regards to delivery [26].

Since one of the priorities announced by the Ministry of health in the health transformation plan to lower maternal as well as fetal complications and mortality is reducing nonessential C-sections, and one of the nonmedical reasons of cesarean in Iran is fear [27], the findings of the present study regarding labor and delivery recommend that provision of suitable psychological and physical atmosphere and developing both pharmacological and nonpharmacological pain alleviation methods in order to establish a sense of security and relaxation among pregnant women should be incorporated in the policy making and management of healthcare centers [28].

Furthermore, since the existing studies [29–32] concur with the recommendations of the labor and delivery of the guideline proposed by this study with regard to the necessity of involving women in the care process, women’s awareness should be raised about care and intervention. In fact, they should be involved in clinical decision-making [29] so that parturient women can act freely [30]. Moreover, frequent vaginal examinations without no consent and no proper justification of the parturient women should be avoided [4], and the privacy as well as the religious and cultural concerns of patients should be absolutely respected [31]. The panel of experts also had a consensus in this regard and stated that frequent vaginal examinations should be avoided without acquiring permissions or proper justification of the parturient women. Tanaka et al. [32] concluded that the number of examinations were more than 40% (5 and more) and vaginal examinations were 1.5 times more at night than daytime [32].

Healthcare providers should also provide their services in a friendly and respectful space. They should regard women’s needs, values, and preferences as their main axes of care. They should also be persistent as well as responsive about the problems in any periods of pregnancy, delivery and postpartum.

The insufficient speed of care providers in crowded delivery rooms as well as their heavy workload can adversely affect the maternal experience of delivery [33]. In fact, psychological problems and occupational burnout of staff can directly affect their working spirit and their behavior toward sympathy with mothers [34]. Thus, the present guideline, regarding occupational and educational issues, recommend that the care provider team should be trained in professional ethics. The hospital government system should also have an atmosphere filled with respect to provide the staff with occupational, financial, and psychological security.

The findings of this study indicated the roles of various factors such as management systems, policymaking, healthcare systems, socioeconomic status, pregnancy status, and the cultures and attitudes of women and care providers in respectful maternity care. Each factor can be analyzed in future studies.

One of the main strong points of this study was that here in addition to applying quantitative and qualitative results, the opinions of a specialist panel were used who were active at different universities across Iran, which can hence increase the generalizability of the results to the entire country. Regarding the limitations, some of the recommendations presented in the present study have been provided considering the cultural context and conditions of Iran. However, other countries and cultures can localize the presented guideline given the conditions, culture, and laws of their own country, and benefit from the results.

## Conclusion

The guideline obtained from this research with regards to RMC can be employed for management, policymaking, educational, and research purposes, and be effective in enhancing the quality of services provided to pregnant mothers. Considering the increasing rate of C-section in Iran, it is expected that with implementing of guideline, women would have greater tendency to undergo natural delivery. Further, implementation of the guideline will help gain awareness about the benefits and obstacles of executing this guideline across the Iranian population. Indeed, we could also provide this guideline to policymakers and planners, so that they would use it as a standard for improving RMC. Nevertheless, in this regard the grounds for implementing national protocols of respectful maternal care such as human and financial resources as well as hospitalization environment should be provided. Since there is no comprehensive guideline in WHO or presented by other countries for RMC, this study was designed to compose RMC guideline in Iran based on a multiphase study. This guideline can be also considered in other parts of the world as well.

## Data Availability

The datasets used and/or analysed during the current study available from the corresponding author on reasonable request.

## Abbreviations

D&A: Disrespect and abuse
RMC: Respectful maternity care
WHO: World Health Organization
AGREE: Appraisal of Guideline Research and Evaluation
